# The FN400 Tracks the Frequency of Task‐Irrelevant Distractors in the Environment

**DOI:** 10.1111/psyp.70392

**Published:** 2026-09-10

**Authors:** Seth A. Marx, Geoffrey F. Woodman

**Affiliations:** ^1^ Department of Psychology, Vanderbilt Vision Research Center Vanderbilt University Nashville Tennessee USA

## Abstract

The frontal N400 (FN400) is an event‐related potential (ERP) typically observed approximately 300–500 ms post‐stimulus and characterized by less negative (more positive) amplitudes for previously encountered stimuli relative to novel stimuli. Here we sought to determine what other statistics of the environment the FN400 might track. Some theories of human memory propose that contextual elements are bound with our representations of targets in our episodic representations. If the FN400 is a mnemonic signal tracking these target‐to‐contextual associations, then it should also respond differently to novel versus previously encountered task‐irrelevant distractors that surround targets. We tested this hypothesis using a visual search task in which we could finely manipulate distractor frequencies to determine how closely the FN400 tracked the statistics of these contextual elements. Across two experiments, we manipulated how many times task‐irrelevant stimuli were shown to test the possibility that the FN400 tracks the familiarity of the task‐irrelevant context. In Experiment 1, the frequency of the distractors was varied parametrically in a visual search task by embedding 0–3 previously encountered distractors in search arrays. The FN400 activity became increasingly positive as the number of repeated distractors increased. In Experiment 2, we asked whether this distractor sensitivity was fine enough to detect a single repetition of just one distractor. We found that this minimal repetition reliably produced a more positive FN400 than all‐novel distractor displays. These results show that FN400 amplitude provides a highly sensitive signal regarding the statistics of the task‐irrelevant context, providing new information about the details of the episodes to which this memory component has access.

## Impact Statement

By directly manipulating the frequency of task‐irrelevant stimuli, this study provides novel evidence that the FN400 is sensitive to changes in the statistics of task‐irrelevant elements in the environment. These findings contribute new observations to a long‐standing debate regarding whether the FN400 indexes a continuous familiarity signal that includes task‐irrelevant contextual information, such as the frequency of repetition of distractors in a visual search array.

## 1 Introduction

Electroencephalography (i.e., EEG) and the averaged event‐related potentials (i.e., ERPs) provide a way of eavesdropping on the brain as it attends to and stores information in memory (Woodman 2010), with one of the more useful components of subjects' ERP waveforms for tracking memory storage being the FN400 (Rugg et al. 1998). It was discovered by having participants study words and then testing the participants' memories by recognizing words as old (from the word list) versus new (not from the word list). The FN400 was shown to be more sensitive than the participants' behavioral reports because it distinguished between previously seen words that were unrecognized compared to new test words (Rugg and Curran 2007; Voss and Paller 2009). That is, even when the participant could not report the presence of a memory, their FN400 could nonetheless report that an old item was present (Rugg et al. 1998). This has resulted in the common view that the FN400 indexes a familiarity detection mechanism (Rugg and Curran 2007), likely due to process fluency being attributed to familiarity (Bader et al. 2023; Mecklinger and Bader 2020), or conceptual fluency given the context (Leynes and Mok 2020; Paller et al. 2012). Generally, this ERP component has been studied using isolated words or pictures to elicit the waveform (Rugg and Curran 2007; Rugg et al. 1998; Servant et al. 2018; Williams et al. 2025), leaving open the possibility that it is a memory potential that keeps track of task‐irrelevant contextual information, in addition to the tracking of target familiarity that has already been shown. As we just reviewed, we know that the FN400 is sensitive enough to track how well an item has been encoded from a single‐shot learning event (Voss and Federmeier 2011), as well as the effects of the accumulation of exposures to targets subjects are trying to memorize (Servant et al. 2018), even when subjects cannot remember those target items. However, far less is known about the sensitivity of this component to the task‐irrelevant information available in the environment. Existing experiments have shown that the FN400 is sensitive to task context (Bader et al. 2023), whether new and old items are blocked or intermixed (Leynes and Upadhyay 2022), and whether a memory target is surrounded by the same context picture at encoding and test (Tsivilis et al. 2001). In the present study, we wanted to test the possibility that the FN400 precisely tracks the memory storage of task‐irrelevant distractor information.

Previous empirical studies of the FN400 using recognition memory tests suggest that the amplitude of this component can be affected by contextual elements (Leynes and Mok 2020). Most relevant for the present study, Tsivilis and colleagues showed that amplitudes of the FN400 were sensitive to seeing a central to‐be‐remembered picture surrounded by different irrelevant contextual pictures (Tsivilis et al. 2001). This design presented the visual system and brain with a large manipulation of context, in which vast swaths of the visual system would have had different inputs across the same versus different context manipulations. Other studies have sought to test for contextual influences using stimuli that people have seen a different number of times during their lives, with their entire life being the relevant context. These studies generally show an increasingly positive FN400 amplitude for stimuli with higher versus lower lifetime frequencies, although these subtle effects co‐occur with larger modulations due to stimulus and conceptual repetitions, changes based on the task being performed, and other within‐experiment factors which can obscure this phenomenon (Bader et al. 2023; Ecker et al. 2007; Leynes and Mok 2020; Mecklinger and Bader 2020). Here, we wanted to test the sensitivity of the FN400 to changes in memory context using a manipulation in which the low‐level sensory energy from the stimuli that formed these contextual elements was matched across trials.

Our questions regarding the sensitivity of the FN400 to task‐irrelevant context are driven by models of human memory that propose that we use contextual representations to store information, with these contextual details providing retrieval cues to be accessible later when memory is searched (Polyn et al. 2009). If the FN400 indexes an episodic memory mechanism, then we would expect the FN400 to be related to the storage of task‐relevant and irrelevant information. Alternatively, the FN400 may not index a mechanism of memory related to the contextually rich episodic memories that models have proposed, but instead measures target focused representations, such as those stored in working memory (Carlisle and Woodman 2019; Olivers et al. 2011).

In Experiment 1, we developed a visual search paradigm in which the statistics of the task‐irrelevant environment was parametrically manipulated by repeating distractor objects presented on the screen with a target a different numbers of times across trials. To create contextual arrays with different task‐irrelevant scene statistics that we could precisely quantify, we manipulated how many repeated distractors were presented on each trial, ranging from displays containing zero repeated distractors (i.e., only novel items) to arrays in which all three distractors were repeated frequently across trials. If the FN400 is sensitive to the task‐irrelevant context of the environment, then mean amplitudes should become progressively more positive as the number of repeated distractors in the search array increases.

In Experiment 2 we provided a more sensitive test of the proposal that the FN400 might track task‐irrelevant context. We did this by reducing the possible environmental repetition to a single task‐irrelevant object repeating just once from the immediately preceding trial. If the FN400 tracks the statistics of the task‐irrelevant context, then this minimal dose of repetition should modulate the FN400 amplitude. In both Experiment 1 and 2, the statistics of the task‐relevant objects were identical whether distractors were repeated or not, meaning any FN400 effects that we observe across our manipulation of the task‐irrelevant context cannot be explained by different target processing demands.

## 2 Experiment 1

The goal of Experiment 1 was to test the possibility that the FN400 tracks the statistics of the task‐irrelevant context, not just task‐relevant information. To this end, we presented search arrays that had one of two possible targets in each array and had subjects reported which target was present on each trial with a button press. Each array of objects contained one target and three distractor objects. These distractors could be novel pictures of objects not shown to the subject before, or they could be from a set of randomly selected object pictures that were repeated frequently across trials. By presenting 1, 2, or 3 of these repeating distractors, we were able to create a parametric manipulation of the context statistics. We know that the FN400 clearly tracks the statistics of target information and here we provided a novel test of whether this key memory effect also tracks the statistics of the context, although this information is not relevant for the simple search task being performed.

### 2.1 Methods

#### 2.1.1 Subjects

Eighteen (14 female) participants with normal color vision and normal or corrected‐to‐normal visual acuity were recruited and consented for Experiment 1. Informed written consent for procedures was approved by the Vanderbilt University Institutional Review Board. Each participant was compensated with course credit or $20 per hour for their time.

#### 2.1.2 Stimuli

**FIGURE 1:**
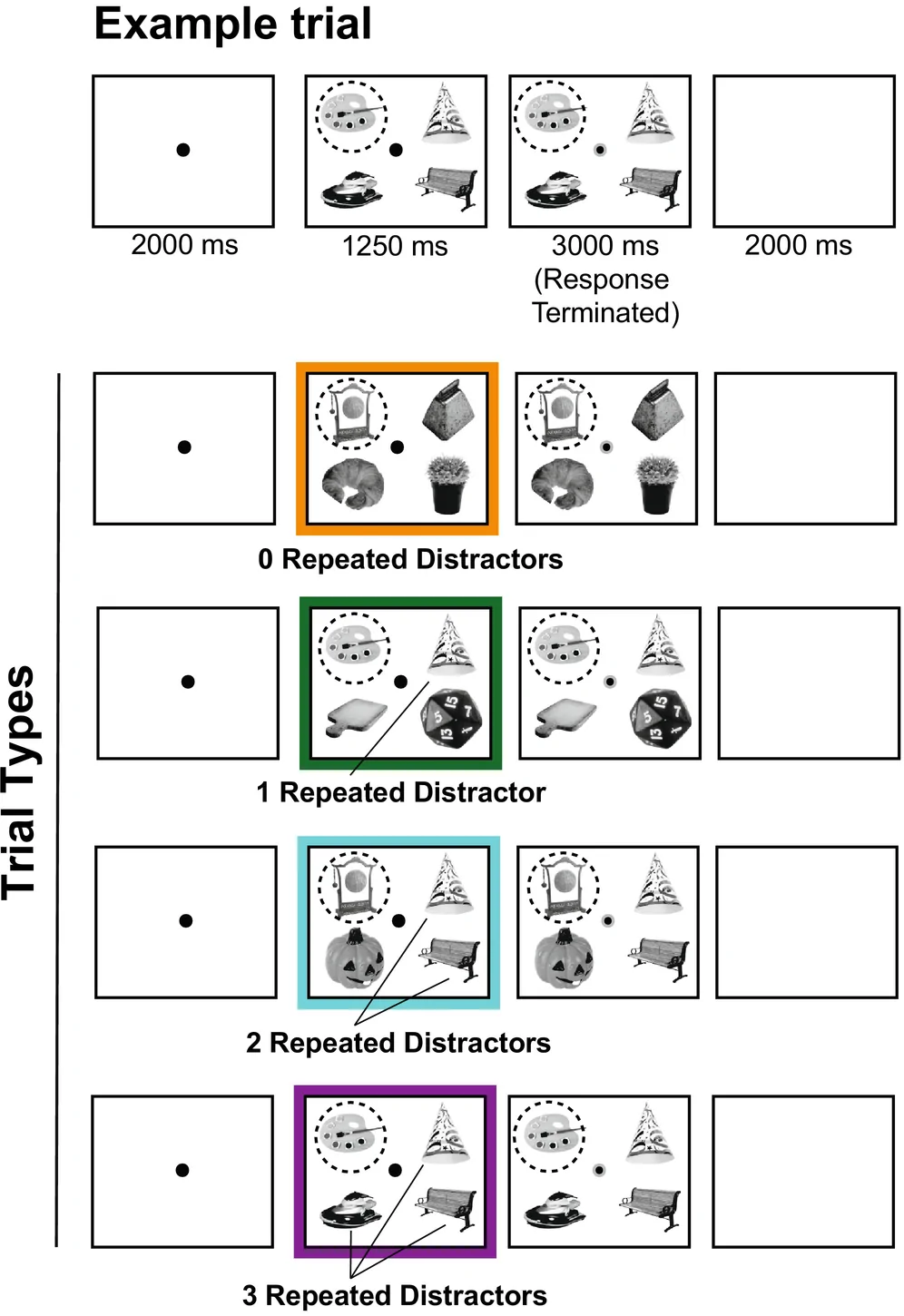
An example trial sequence from Experiment 1. After fixation, four grayscale objects were presented simultaneously in fixed quadrant locations. One item was the target (A) or (B), and the remaining items were distractors. The number of repeated distractors in the array varied across trials (0, 1, 2, or 3 repeated distractors).

Participants were instructed to fixate on a central black dot (1° × 1° in visual angle, 0.03 cd/m^2^) presented on a white background (38.2 cd/m^2^) throughout each trial of the experiment, as shown in Figure 1. Trials began with a 2000 millisecond (ms) fixation. Next, we presented four images (each 5° × 5° of visual angle) from a set of objects (Brady et al. 2008) in four stable locations centered in the four quadrants for 1250 ms. All images were grayscale to reduce the guidance of attention driven simply by color. The fixation dot lightened to gray (24.9 cd/m^2^), indicating when participants could respond. By not allowing participants to respond until they had viewed the array for 1250 ms we were able to measure subjects' memory‐related waveforms uncontaminated by response selection activity. Trials were then response‐terminated with a maximum search time of 3000 ms and followed by a 2000 ms inter‐trial interval with a blank screen.

Two random stimuli were selected as target “A” and target “B”, respectively. Three random stimuli were selected as repeated distractors, and the rest of the stimuli were compiled into a pool of novel distractors. For each subject, the A and B target, repeating distractors, and novel distractors were randomly resampled from the pool. Subjects completed 960 trials, 240 trials for each of the distractor conditions (0, 1, 2, or 3 previously seen distractors). Across all trials, target “A” and target “B” were evenly distributed as the target in the search (50% of each type randomly interleaved). Additionally, the old distractor set sizes were randomly distributed across trials. Specifically, there were four trial types that varied the number of previously‐seen distractors—3, 2, 1, and 0—where the fourth stimulus presented was either target A or target B.

#### 2.1.3 Procedure

Participants were instructed to respond as fast and as accurately as possible using the provided Logitech Precision Gamepad (Lausanne Switzerland; Vendor ID: 1133, Product ID: 49690). Participants responded to whether target “A” was present using the “5” button or whether target “B” was present using the “6” button, which were the left and right index finger trigger buttons on the gamepad.

#### 2.1.4 EEG Recording and Processing

EEG data were recorded using the international 10–20 electrode sites (Fz, Cz, Pz, F3, F4, C3, C4, P3, P4, PO3, PO4, O1, O2, T3, T4, T5, and T6) along with single electrodes on the left mastoid, right mastoid, left 1‐cm lateral to the external canthi, right 1‐cm lateral to the external canthi, and below the right eye (Electro‐cap International Inc., Eaton, OH). The right mastoid was the online reference during the acquisition. The equipment amplified the signals with a gain of 20,000, applied a band‐pass filter from 0.01 to 100 Hz, and digitized the data at a sampling rate of 250 Hz (SA Instruments Inc., San Diego, CA).

Subject EEG data sets were processed using EEGLAB (Delorme and Makeig 2004) and ERPLAB (Lopez‐Calderon and Luck 2014). Prior to processing, EEG data were re‐referenced to the average of the left and right mastoids, excluding the vertical eye movement electrode and horizontal eye movement electrodes.

The continuous EEG data were segmented into epochs corresponding to individual trials, time‐locked to the stimulus presentation. The epoch window ranged from −200 to 1000 ms, with the data baseline‐corrected from −200 to 0 ms. Epochs were binned by trial type. Epochs were rejected if vertical eye movements exceeded +/− 75 μV or if any channel remained flatlined between −2 μV and 2 μV. Additionally, to account for between‐subject variation in saccades, thresholds were adjusted for each participant such that the initial threshold was +/− 24 μV (based on visual saccade inspections) and increased no more than +/− 40 μV greater than/lesser than the original threshold. Datasets were used in the analyses if there were at least 75% of trials correct and accepted for each main trial type of each experiment. Artifact‐rejected epochs and trials with incorrect responses were removed from all analyses.

#### 2.1.5 ERP Analyses

Continuous EEG data were segmented into stimulus‐locked epochs and averaged separately for each experimental condition to create event‐related potentials (ERPs). Mean amplitudes are shown from the frontal midline electrode (Fz), where the FN400 was maximal, and following norms in the literature (Rugg and Curran 2007; Servant et al. 2018). For the statistical analyses, mean amplitudes were calculated across three levels of electrode position (frontal, central, and parietal). Each level consisted of the average of the midline electrode and its adjacent left and right electrodes (frontal: F3, Fz, F4; central: C3, Cz, C4; parietal: P3, Pz, P4). Based on prior literature, we defined the FN400 analysis window as 300–500 ms post‐stimulus onset and report the statistical results of this time window. However, based on visual inspection of the grand‐average waveforms we confirmed the same results are obtained with a longer 200–800 ms window, although this window picked up more response selection and preparation activity. Mean voltage within the 300–500ms interval served as the dependent measure for all statistical analyses. Statistical analyses were conducted using JASP version 0.19.1. For display purposes, we show filtered data (30 Hz half‐power cutoff), but the analyses were performed on unfiltered waveforms. We used all available electrodes in our montage for our voltage maps, shown in the data figures for purely illustrative purposes.

Post hoc power analyses were performed using G*Power (Faul et al. 2007) to assess whether we had sufficient power to study the effect of interest. It indicated that the observed effect of distractor repetition (*η* ^2^_p_ = 0.639; *f* = 1.33) was associated with high statistical power (1 − *β* = 1.00) at *α* = 0.05 with *N* = 17, and the planned analysis across distractor repetition levels corresponded to a large within‐subject effect size (*d*_*z*_ = 1.75). Using *α* = 0.05 and *N* = 17, the analysis indicated high statistical power (1 − *β* = 1.00). We performed similar power analyses for Experiment 2. The post hoc power analysis of the comparison between all‐new distractor trials and one‐repeated‐distractor trials indicated an observed effect size (*η* ^2^_p_ = 0.333; *f* = 0.475), *α* = 0.05, and *N* = 14, measured with high statistical power (1 − *β* = 0.91).

### 2.2 Results

Behavioral data showed no evidence that distractor repetitions influenced performance. Accuracy was unchanged across conditions: 3 Repeated Distractors (*M* = 0.992, SD = 0.013); 2 Repeated Distractors (*M* = 0.990, SD = 0.012); 1 Repeated Distractor (*M* = 0.993, SD = 0.009); 0 Repeated Distractors (*M* = 0.992, SD = 0.009). There was no significant effect of Repeated Distractors, *F* (3, 48) = 0.472, *p* = 0.704, *η* ^2^_p_ = 0.029.

Response times (RTs) were stable across conditions: 3 Repeated Distractors (*M* = 357.150 ms, SD = 111.176 ms); 2 Repeated Distractors (*M* = 354.052 ms, SD = 113.818 ms); 1 Repeated Distractor (*M* = 360.186 ms, SD = 116.760 ms); 0 Repeated Distractors (*M* = 360.167 ms, SD = 115.404 ms). There was no significant effect of Repeated Distractors, *F* (3, 48) = 0.814, *p* = 0.493, *η* ^2^_p_ = 0.048. Bayesian analyses supported the null hypothesis, providing substantial evidence against an effect of distractor repetition on either accuracy (BF_01_ = 7.731) or RT (BF_01_ = 5.445). Participants' performance was unaffected by distractor repetition at the behavioral level, such that we might conclude that these distractor items were perfectly filtered out by attention (Vogel et al. 2005). That is, when manipulating distractors has no effect, researchers assume that this information has been filtered out by attention prior to memory encoding (Woodman 2013). Next, we ask whether the FN400 activity is target focused as a result of this filtering, or whether the FN400 maintains contextual elements, as episodic memory theories predict.

**FIGURE 2:**
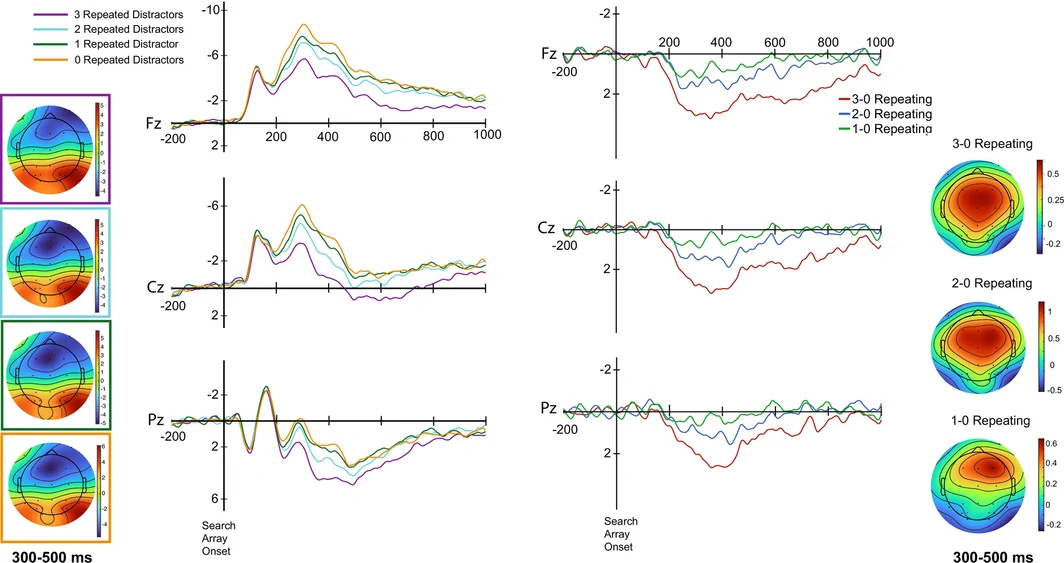
The results from Experiment 1. The leftmost panels show the scalp distributions of the raw waves from each trial type. The middle‐left panels show the grand‐average ERPs at Fz (top), Cz (middle), and Pz (bottom) time‐locked to array onset (baseline −200 to 0 ms). The middle‐right panels show the difference waves in which the 0 repeating distractor arrays serve as the baseline measured during the FN400 window of 300–500 ms post‐stimulus onset. The rightmost panels show the distributions of the difference waves between each trial type and the 0 repeating distractor baseline during this same 300–500 ms window.

As shown in Figure 2, the amplitude of the frontal negativity significantly differed across trial conditions as a function of the number of repeated distractors. A RM ANOVA yielded a significant main effect of Electrode Site (i.e., Frontal, Central, Parietal) on mean frontal positivity amplitudes, *F* (2, 32) = 41.68, *p* < 0.001, *η* ^2^_p_ = 0.723, and a main effect of Repeated Distractors, *F* (3, 48) = 36.08, *p* < 0.001, *η* ^2^_p_ = 0.693, as mean amplitudes became less negative as the number of repeated distractors increased. The interaction of Electrode Site × Repeated Distractors was significant in the 300–500 ms window, *F* (6, 96) = 3.51, *p* = 0.004, *η* ^2^_p_ = 0.180, indicating that the repetition effect was largest at frontal channels. The frontal distribution of these effects supports our assumption that these effects are generated by the FN400 and related subsequent memory slow waves that are maximal at frontal electrodes (Fukuda and Woodman 2015).

Post hoc tests clarified what drove these omnibus effects. Collapsing across repetition levels, electrode comparisons showed the expected ordering (Frontal more positive than Central, and both more positive than Parietal; all *p*s < 0.001). Collapsing across electrode sites, repetition‐level contrasts showed that the highest repetition condition (3 repeated distractors) differed reliably from lower levels (e.g., 3 vs. 2, 3 vs. 1, 3 vs. 0), and that some adjacent steps were smaller (e.g., 2 vs. 1 and 1 vs. 0 were not reliably different under correction), which is consistent with a graded‐but‐not‐necessarily‐uniform step size across levels. Critically, the planned polynomial analysis supported the central experimental prediction: a significant linear trend (estimate = −1.725, *t*(16) = −8.631, *p* < 0.001, *d* = −0.478), meaning that each additional repeated distractor produced a reliable incremental increase in FN400 positivity (i.e., a strength‐like familiarity signal).

### 2.3 Discussion

Experiment 1 examined whether the FN400 reflects only the storage of task‐relevant information or whether it also indexes task‐irrelevant information derived from a distracting context that can be ignored in the task. We parametrically manipulated the number of repeated distractors within visual search arrays while targets and task demands remained unchanged. Based on subjects' behavior, we could conclude that they successfully filtered out these task‐irrelevant distractors. Behavioral performance was similarly fast and accurate across repetition conditions, indicating that distractor repetition neither facilitated nor interfered with target processing.

Despite behavioral performance suggesting the distractors were filtered out of the processing stream, FN400 amplitudes increased systematically as the number of repeated distractors increased in each array. The amplitude of the FN400 component (i.e., the positivity) scaled with the cumulative repetition of distractors in the array. Because the repeated items were task‐irrelevant and uninformative for target identification, the modulation is unlikely to be explained by intentional encoding. Instead, these recognition judgments were likely taking place automatically (Caerwinski et al. 1992; Logan 2002; Pribram and McGuiness 1975). In sum, the results indicate that the FN400 reflects contextual information present in the perceptual environment, even when subjects are essentially trying to ignore this information.

Importantly, the frontal distribution of the effect supports its identification as an FN400‐like response (Rugg et al. 1998). The graded modulation we observed suggests that the component tracks the statistics of multiple elements in the environment simultaneously. Each repeated distractor contributed incrementally to the overall positivity, consistent with interpretations of the frontal positivity as providing a metric of memory strength (Fukuda and Woodman 2015). Thus, Experiment 1 provides parametric evidence that the FN400 tracks the task‐irrelevant contextual information rather than being restricted to representations of goal‐relevant items.

## 3 Experiment 2

In Experiment 1, we showed that mean FN400 amplitudes were yoked to the number of repeated distractors present in a given search array. In Experiment 2, we asked how sensitive the FN400 would be to task‐irrelevant context changes that are far more subtle. To test this, we used the same experimental paradigm as in Experiment 1, with one key modification: on half of the trials, all distractors were unique, while on the other half of trials, one distractor was repeated from the set of distractors from the immediately preceding trial.

### 3.1 Methods

#### 3.1.1 Subjects

Fourteen participants (11 female) with normal color vision and normal or corrected‐to‐normal visual acuity were recruited and consented for Experiment 2. Informed written consent for procedures was approved by the Vanderbilt University Institutional Review Board. Each participant was compensated with course credit or $20 per hour for their time.

#### 3.1.2 EEG Analysis and Processing

EEG analysis and processing were identical to those of Experiment 1.

#### 3.1.3 Stimuli and Procedure for Experiment 2

**FIGURE 3:**
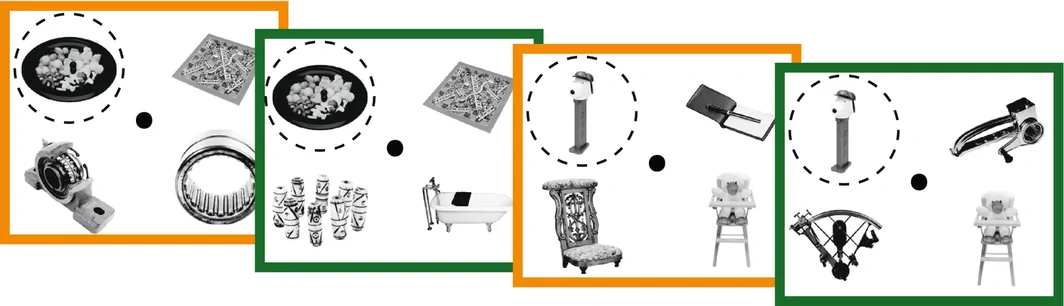
An example trial sequence from Experiment 2. Odd‐numbered trials contained all‐novel distractors; on even‐numbered trials, one distractor was repeated from the immediately preceding odd‐numbered trial (with repeated‐item location counterbalanced relative to the target).

The procedure for Experiment 2 was identical to Experiment 1, as shown in Figure 3. Two random stimuli were selected as targets “A” and “B”, respectively. For each odd‐numbered trial, three new distractors were randomly selected and randomly placed in the remaining three quadrants. For each even‐numbered trial, one distractor from the previous odd trial was randomly selected and repeated in the current trial. For 50% of the even trials, the repeated distractor appeared on the same side as the target, while it appeared on the opposite side of the target the other 50% of the time. Target quadrants were counterbalanced across trials. Subjects completed 576 trials total, 288 per condition.

#### 3.1.4 ERP Analyses

ERP analyses were identical to those of Experiment 1.

### 3.2 Results

Behavioral results indicated that participants responded quickly and accurately to both trial types with no significant difference in performance as a function of whether one of the distractors had repeated once. Mean accuracies were stable across trial conditions: all new distractors (*M* = 0.995, SD = 0.008); 1 repeated distractor (*M* = 0.995, SD = 0.005). Mean RTs were also stable across trial conditions: all new distractors (*M* = 336.296 ms, SD = 97.118 ms); 1 repeated distractor (*M* = 335.188 ms, SD = 94.251 ms). There was no significant effect of repeated distractors on accuracy, *F* (1, 13) = 0.305, *p* = 0.590, *η* ^2^_p_ = 0.023, nor response time (RT), *F* (1, 13) = 0.134, *p* = 0.720, *η* ^2^_p_ = 0.010. Bayesian analyses using the same factor of Repeated Distractor provided moderate evidence for the null hypothesis (Accuracy BF_01_ = 2.595; RT BF_01_ = 2.739), indicating that participants' performance was unaffected by distractor repetition at the behavioral level.

**FIGURE 4:**
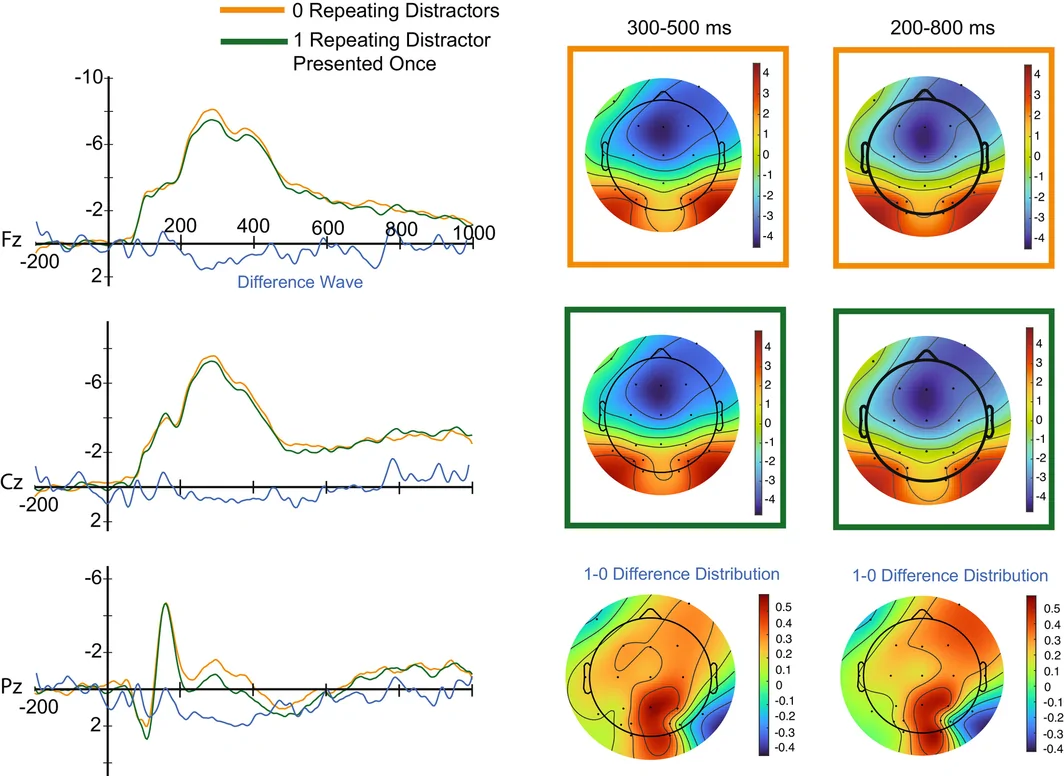
Results from Experiment 2. Left panels show the grand‐average ERPs at Fz (top), Cz (middle), and Pz (bottom) time‐locked to array onset (baseline −200 to 0 ms). The right panels show the scalp topography of the repetition effect (averaged over the 300–500 ms FN400 window), as well as the difference wave distribution.

Repeating an item modulated the FN400 amplitude as shown in Figure 4. An ANOVA yielded a significant main effect of Electrode Site (i.e., Frontal, Central, and Parietal), *F* (2, 26) = 75.51, *p* < 0.001, *η* ^2^_p_ = 0.851, and of Repeated Distractors on amplitude, *F* (1, 13) = 6.97, *p* = 0.020, *η* ^2^_p_ = 0.349. However, the ANOVA did not yield a significant Electrode × Repeated Distractors interaction, *F* (2, 26) = 0.340, *p* = 0.715, *η* ^2^_p_ = 0.025, due to parietal electrodes showing the amplitude effect of repetition as well. This could be due to overlap with the P3b, but it is notable that the behavior did not show evidence of the distractor repetition effect.

Preplanned tests of frontal amplitudes did yield a significant main effect of Repeated Distractors, *F* (1, 13) = 6.498, *p* = 0.024, *η* ^2^_p_ = 0.333, indicating that trials with one repeated distractor had a significantly more positive mean frontal positivity amplitude than trials with all novel distractors, consistent with the prediction from the context hypothesis.

### 3.3 Discussion

Experiment 2 provided a finer‐grained test the hypothesis that the FN400 tracks task‐irrelevant context by introducing the minimal possible repetition: a single distractor repeated from the immediately preceding trial. As in Experiment 1, behavioral performance was unaffected, again demonstrating a dissociation between neural familiarity signals and overt performance. Behaviorally, we would conclude that this once repeating distractor had been filtered out of the processing stream.

Nevertheless, a reliable FN400 modulation was observed at frontal sites, with greater positivity for arrays containing a single repeated distractor compared to all‐novel distractor object arrays. This finding demonstrates that the FN400 responds not only to accumulated repetitions but also to minimal changes in the statistics of the context. A single distractor element in the scene was repeated, and this was registered in this long‐term memory potential. These findings demonstrate that the memory storage mechanism indexed by this potential stores both task‐relevant objects and also carries information about task‐irrelevant contextual elements in our visual world.

## 4 General Discussion

The present study examined whether the FN400 reflects experience with task‐relevant items or whether it indexes experience derived from the broader context. Across two experiments, we manipulated the frequency of task‐irrelevant distractors while target processing and task demands remained constant. FN400 amplitudes varied systematically with distractor repetition despite no changes in behavioral performance, demonstrating that the component tracks memory for task‐irrelevant contextual information.

Our two experiments provide converging evidence across different time scales of contextually repeating elements. We saw a graded FN400 amplitude increase with distractors experienced across the long term and sensitivity to a single recent exposure across the short term. These properties indicate that the component does not merely distinguish old from new items or reflect conceptual fluency but instead can track contextual elements that can support rich episodic memories. Context was manipulated directly by repeating irrelevant distractors embedded within visual search arrays, as used in the cognitive literature (Woodman and Chun 2006). Experiment 1 revealed a robust linear increase in FN400 amplitude as the number of repeated distractors increased, indicating a graded signal that accumulates across the elements in the visual field, representing the task‐relevant object and irrelevant statistics of the contextual elements. Experiment 2 demonstrated that even a single repetition from the immediately preceding trial was sufficient to modulate the component. Together, these findings show that the FN400 is sensitive to both accumulated exposure and single doses of repetition in the task‐irrelevant context, consistent with contextual models of memory that propose target and distractor information is encoded into long‐term memory together (Polyn et al. 2009). In these models, task‐relevant objects and task‐irrelevant objects occupy a contextual representation that encodes the current state of the brain to episodic memory. These contextual details later aid memory retrieval of the event (Kragel and Voss 2021). The interpretation of the current findings that we currently favor due to parsimony is that the FN400 indexes this streaming of current context to episodic memory.

A couple of our design decisions and the accompanying results deserve discussion. First, we used a visual search task, not an explicit memory task in which we asked subjects to report information from memory about targets or the distractors that we manipulated. We did this on purpose because we wanted the distractor objects to be truly task irrelevant. However, it would be useful for future experiments to relate the FN400 amplitude modulations due to changes in context, particularly to those that aid later memory retrieval, as the additional complexity revealed here should confer a behavioral advantage for matching contexts compared to remembering information across changing contexts (see Mecklinger and Bader 2020, for a thorough discussion of this issue). Second, we had our subjects view the visual search array for just over 1 s before being cued to report which target was present with a button press on each trial. This was done to prevent component overlap from waveforms related to response selection and execution from overlapping with the FN400 waveform elicited by the search array. However, in ERP studies the possibility of component overlap is ubiquitous (Luck 2005), and our own results show that when responses were ~30 ms faster in Experiment 2, we observed more component overlap between the FN400 and the P3b related to pressing the button to respond. Thus, it is possible that the target elicits a stable FN400 and the contextual modulations of the FN400 that we are seeing here are due to component overlap. The idea is that the distractor statistics elicit separate components that then overlap with the FN400 in time and space across the scalp. This intriguing possibility will require future experiments and combinations of methods to address, but the necessity of this work is illustrated by the following empirical results.

Our current empirical results align with findings from Ecker et al. (2007), but they interpreted their effects as due to overlapping brain responses. They found that mean FN400 amplitudes were significantly greater when attention was distributed across multiple stimuli (i.e., a familiar target object on a familiar background), compared to when attention was directed solely to a target object without the background. The more positive‐going waveform was proposed to reflect a conjunctive signal of the combined familiarity strength of multiple items. Thus, both of these results could be due to distractor objects eliciting a positive potential and overlapping with the FN400, as future research will be able to determine. Our approach in this study was to view the amplitude modulations elicited across frontal‐central electrodes during our memory tasks as the memory‐related FN400. This approach is one of lumping together observations in which better memory follows a larger amplitude positive deflection of the frontal slow waves (Fukuda and Woodman 2015), with the theoretical view that the FN400 and the N400 waveforms are elicited by the same underlying memory processes (Voss and Federmeier 2011). This is not the only view of these memory‐related effects. Mecklinger and Bader (2020) provide a thorough review of the perspective that the FN400 is driven by episodic memory processes sensitive to familiarity, while the N400 is driven by semantic memory processes also sensitive to familiarity. Under this latter view, the present findings provide further support for a perspective in which the FN400 is related to episodic memory as it tracked the task‐irrelevant context, due to the frontal distribution we observed.

In summary, the present findings broaden the functional interpretation of the FN400. Rather than signaling memory only for task‐relevant items, the component indexes memory for task‐irrelevant contextual information. One theoretical perspective on these findings is that they are consistent with the view that no information is ever truly irrelevant for memory, as the context surrounding a task‐relevant object is stored with that object representation, and we can see the effect of this storage because the irrelevant objects can work as retrieval cues (Kahana 2012). Thus, the present findings support the idea that the FN400 measures what is stored in our contextually rich episodic memory.

## Author Contributions

**Seth A. Marx:** visualization, methodology, investigation, writing – original draft, formal analysis, data curation, validation, software. **Geoffrey F. Woodman:** conceptualization, funding acquisition, writing – review and editing, project administration, supervision, formal analysis, software, resources.

## Funding

This work was supported by the National Science Foundation (BCS‐2147064), National Eye Institute (T32‐EY007135).

## Conflicts of Interest

The authors declare no conflicts of interest.

## Data Availability

The data that support the findings of this study are openly available in OSF at https://osf.io/zmvw3/, reference number zmvw3.
